# The effectiveness of high-flow nasal cannula during sedated digestive endoscopy: a systematic review and meta-analysis

**DOI:** 10.1186/s40001-022-00661-8

**Published:** 2022-02-24

**Authors:** Yu-Xin Zhang, Xing-Xiang He, Yu-Ping Chen, Shuai Yang

**Affiliations:** 1 Department of Gastroenterology, Zhuhai People’s Hospital (Zhuhai Hospital Affiliated with Jinan University), Zhuhai, 519000 China; 2 Department of Gastroenterology, The First Affiliated Hospital of Guangdong Pharmaceutical University, Guangzhou, 510080 China; 3 Department of Emergency Intensive Care Unit, Zhuhai People’s Hospital (Zhuhai Hospital Affiliated with Jinan University), 79 Kangning Road, Xiangzhou District, Zhuhai, 519000 China

**Keywords:** High-flow nasal cannula oxygen (HFNC), Digestive endoscopy, Sedation, Hypoxemia, Airway intervention

## Abstract

**Background:**

Studies evaluating the role of high-flow nasal cannula (HFNC) in sedated digestive endoscopy have reported conflicting results. This meta-analysis evaluates the effectiveness of HFNC in patients undergoing digestive endoscopy procedures under sedation.

**Methods:**

PubMed, Medline, EMBASE, Cochrane Central Register of Controlled Trials and Web of Science, were searched from inception to 31 July 2021. Only randomized clinical trials comparing HFNC with standard nasal cannula oxygen (SNC) during sedated digestive endoscopy were included. The incidence of hypoxemia was the primary outcome, and the secondary outcome was the percentage of patients who needed airway interventions.

**Results:**

Seven studies that enrolled 2998 patients were included. When compared to SNC, HFNC was associated with a significant reduction in hypoxemia incidence (OR 0.24, 95% CI 0.09 to 0.64) and airway intervention requirements (OR 0.15, 95% CI 0.03 to 0.69), with both high heterogeneity (I^2^ = 81% and 94%). In subgroup analysis, when hypoxemia was defined as pulse oxygen saturation (SpO_2_) < 90%, low risk of hypoxemia subjects who received HFNC were associated with a significant reduction in hypoxemia incidence (OR 0.02, 95% CI 0.00 to 0.07; heterogeneity I^2^ = 39%) and airway intervention requirements (OR 0.02, 95% CI 0.01 to 0.04; heterogeneity I^2^ = 15%). However, in the high risk of hypoxemia subjects, there were no significant differences between the two oxygen administration techniques in both primary (OR 0.81, 95% CI 0.36 to 1.78; heterogeneity I^2^ = 0%) and secondary outcomes (OR 0.85, 95% CI 0.46 to 1.59; heterogeneity I^2^ = 0%).

**Conclusions:**

Compared to SNC, HFNC not only reduce the incidence of hypoxemia but also reduce the requirements for airway interventions during sedated digestive endoscopy procedures, especially in patients at low risk for hypoxemia. In high risk of hypoxemia patients, there were no significant differences between the two oxygen administration techniques.

*Trial registration* PROSPERO International prospective register of systematic reviews on 28 July 2021, registration no. CRD42021264556.

**Supplementary Information:**

The online version contains supplementary material available at 10.1186/s40001-022-00661-8.

## Introduction

In recent years, the number of digestive endoscopy procedures has extended steadily [1, 2]. More than fifty percent of digestive endoscopy procedures are performed under sedation, relying on anesthesia services [3, 4]. The purpose of sedated digestive endoscopy is to relieve patient discomfort and anxiety, provide better quality examination and reduce the patient’s memory of endoscopy experience [5]. However, using common medications such as benzodiazepines and propofol often leads to respiratory depression, airway obstruction, and subsequent hypoxemia [6]. Severe hypoxemia will interrupt the endoscopic procedure, and require immediate airway interventions, such as chin lift, jaw thrust, nasal airway, mask ventilation, invasive or non-invasive ventilation. Continuous hypoxemia may lead to arrhythmia, myocardial ischemia, permanent neurological complications, or death [7–9].

Administering supplementary oxygen using a standard nasal cannula (SNC) is the current standard of care for most patients undergoing sedation for digestive endoscopy procedures [10]. Usually, the highest oxygen flow SNC can provide is 6 L/min. Under this circumstance, inspired oxygen concentration (FiO_2_) in the distal airways is no more than 40% [11]. Higher inspired oxygen concentrations are not possible with SNC, because the patient's inspiratory flow changes with each breath. If the inspiratory flow is greater than the flow of oxygen, then room air is entrained, which lowers the FiO_2_.

High-flow nasal cannula (HFNC) oxygen is a recently developed non-invasive oxygen therapy system. It can provide heated and moist oxygen through the nasal cannula, as well as offer a much higher and predictable gas flow rate (up 60 L/min) and FiO_2_ (up to 100%) [12, 13]. In HFNC therapy, the high flow also produces positive pressure within the nasopharyngeal space and thoracic cavity [14], which reduces airway obstruction and increases the end-expiratory lung volume [15–18]. Because of its potential to improve oxygenation and ventilation, HFNC has been applied in many clinical situations to prevent hypoxemia, such as in awake fiber-optic intubation [19], conscious sedation during bronchoscopy and some dental treatments under intravenous sedation [16, 20]. In addition, a few randomized controlled trials have shown that HFNC could also reduce the risk of hypoxemia during sedated digestive endoscopy [21, 22], but some studies cannot draw the same conclusion [23, 24].

Is HFNC more effective than SNC in reducing the incidence of hypoxemia during sedated digestive endoscopy? Research in this area is new, and up to now, there are no established guidelines. The aim of the present study is to investigate whether HFNC has more advantages than SNC in sedated digestive endoscopy, with particular emphasis on the effects of preventing hypoxia as well as avoiding airway interventions.

## Materials and methods

This meta-analysis was conducted in accordance with the Preferred Reporting Items for Systematic Reviews and Meta-Analyses (PRISMA) statement [25]. The protocol of this review was registered on PROSPERO (Registration Number: CRD42021264556). Ethical consent was unnecessary, because the data needed to support the current meta-analysis was derived from previously published studies.

### Inclusion and exclusion criteria

The studies had to meet the following inclusion criteria: be randomized and controlled, compare HFNC with SNC, include adult patients who underwent sedated digestive endoscopy (including endoscopic retrograde cholangiopancreatography, esophagogastroduodenoscopy, gastroscopy, gastrointestinal endoscopy, and colonoscopy). Obstetrics and pediatric patient studies were excluded.

### Search strategy

We searched the following five electronic databases: PubMed, Medline, EMBASE, Cochrane Central Register of Controlled Trials, and Web of Science from inception to 31 July 2021. We used the search terms (High-flow nasal cannula oxygen) AND (digestive endoscopy) AND (sedation) AND (randomized clinical trials), limited to humans and adults where possible (details were shown in Additional file 1). We reviewed the references lists of articles for other studies to supplement our search.

### Outcome measures

The primary outcome was to investigate whether HFNC versus SNC resulted in a different incidence of hypoxemia at any time point during the procedure. The secondary outcome was the percentage of patients who need airway interventions (including chin lift, jaw thrust, nasal airway, mask ventilation, invasive or non-invasive ventilation).

### Study selection and data extraction

Two researchers (Shuai Yang and Yu-Xin Zhang) independently evaluated the title and abstract of RCTs that were probably eligible. Then the full text was extracted, and the eligibility according to the eligibility criteria was assessed. Any dispute was resolved through discussion with a third reviewer (Xing-Xiang He). Data extraction was performed using a self-designed data collection form. It included the study ID, the first author’s name and publication year, country, endoscopic method, NCT number, primary and secondary outcomes. Subjects would be considered to be of high risk for developing hypoxemia when they were obese (BMI > 30), or had obstructive sleep apnoea syndrome (OSAS), or were American Society of Anesthesiologists (ASA) physical status 3 or 4. If the subjects were BMI < 30, or without OSAS, or were ASA physical status 1 or 2, the subjects were considered relatively low risk for developing hypoxemia.

### Quality assessment

Using the Cochrane Collaboration Risk of Bias tool, two reviewers (Shuai Yang and Yu-Xin Zhang) independently assessed the methodological quality of the selected studies. Any conflicting opinions were resolved by discussion in the presence of a third investigator (Xing-Xiang He). Every included study was assessed for the following sources of bias: selection bias (random sequence generation and allocation concealment), blinding of participants and personnel, blinding of outcome assessment, incomplete outcome data, selective reporting, and other bias.

### Data synthesis and analysis

Statistical analysis of our study was performed with the cochrane systematic review software Review Manager (RevMan, version: 5.4). Odds ratio (OR) with 95% confidence interval (95% Cl) was calculated for the dichotomous outcomes. Heterogeneity was tested by the I^2^ statistics. When I^2^ was greater than 50%, a random-effect model was used, while if there was no substantial heterogeneity (I^2^ < 50%), the fixed-effect model was used. Two-sided tests were used in all analyses, and *P* < 0.05 was considered statistically significant.

## Results

### Study search

**Fig.1:**
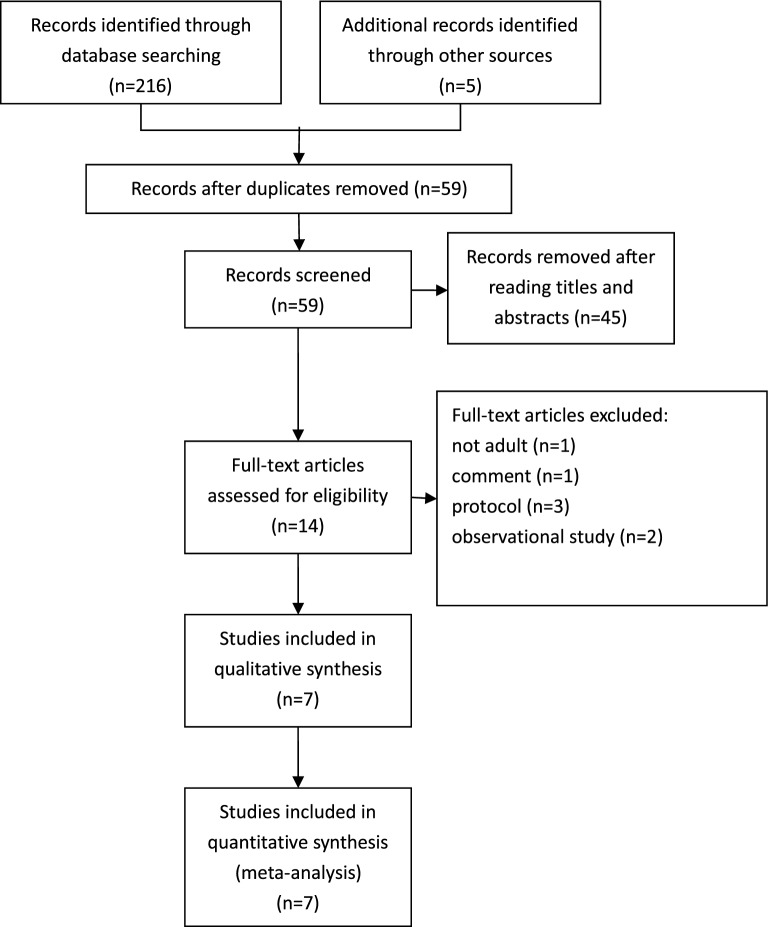
Study flow diagram

Initially, 221 articles were identified (216 were extracted from databases, and 5 were extracted from other sources). After discarding duplicate reports, 59 studies were screened according to their titles and abstracts to determine the possible studies. Finally, the full texts of 14 articles were evaluated, and 7 studies were excluded: 1 was a pediatric patient study, 1 was a comment paper, 3 were protocols, 2 were observational studies. In total, 7 RCTs [21–24, 26–28] were eligible in our meta-analysis, which ultimately included 2998 patients. The flowchart of the study is shown in Fig. 1.

### Study characteristics

The characteristics of individual studies included in this meta-analysis are presented in Tables 1 and 2. These studies were published between 2019 and 2021. Trials took place across North America, Europe, Asia, and Australia. HFNC in two studies was used during ERCP procedural sedation [22, 24], and others were used during esophagogastroduodenoscopy procedural sedation. In 3 of 7 articles, procedures were performed on high risk of hypoxemia subjects [23, 24, 28]. In the other four articles, sedation procedures were performed on low risk of hypoxemia subjects. All studies reported the primary outcome, and 6 of 7 studies reported the secondary outcome [21–24, 26, 28].

**Table 1:** Characteristics of Included Studies

Author (Year)	NCT No.	Country	Population	Endoscopic method	Hypoxemia definition	Hypoxemia risk*	Outcome^#^
Kim 2021 [22]	NCT03872674	Korea	72	ERCP	SpO_2_ < 90%	Low	①②
Teng 2019 [26]	NCT03138850	Taiwan	101	Esophagogastroduodenoscopy	SpO_2_ < 90%	Low	①②
Mazzeffi 2021[27]	NCT03028688	America	262	Esophagogastroduodenoscopy	SpO_2_ < 92%	Low	①
Lin 2019 [21]	NCT03332433	China	1994	Gastroscopy	SpO_2_ < 90%	Low	①②
Nay 2021 [28]	NCT03829293	France	379	Gastrointestinal endoscopy	SpO_2_ < 92%	High	①②
Riccio 2019 [23]	NCT03148262	America	59	Colonoscopy	SpO_2_ < 90%	High	①②
Thiruvenkatarajan 2021 [24]	CTRN12619000397112	Australia	131	ERCP	SpO_2_ < 90%	High	①②

*ERCP* endoscopic retrograde cholangiopancreatography

^*^High: patients were obese (BMI > 30), or had obstructive sleep apnoea syndrome, or were ASA physical status 3 or 4; Low: others

^#^Outcome include: ①Hypoxemia; ②Need for airway interventions

**Table 2:** Patient characteristics of participants in studies included in the meta-analysis

Study, group, %*														
	Kim [22]		Teng [26]		Mazzeffi [27]		Lin [21]		Nay [28]		Riccio [23]		Thiruvenkatarajan [24]	
Characteristic	HFNC *n* = 36	SNC *n* = 36	HFNC *n* = 50	SNC *n* = 51	HFNC *n* = 132	SNC *n* = 130	HFNC *n* = 994	SNC *n* = 1000	HFNC *n* = 191	SNC *n* = 188	HFNC *n* = 28	SNC *n* = 31	HFNC *n* = 65	SNC *n* = 66
Age, mean yr	65.3	67.3	46.7	51.6	62	62	48	47	64$	64$	54	59	69.1	65.5
Male sex	61.1	69.4	38	43.1	87	71	41.5	41	57	51	14	13	43.1	40.9
Operation														
ERCP	100	100			23.5	20.8							100	100
EGD			100	100	76.5	79.2								
gastroscopy							100	100	18.3	20.2				
colonoscopy									52.4	48.9	100	100		
combined^#^									29.3	30.9				
BMI, mean	23.1	22.1	22.5	23.4	28.3	28.2	22.8	23	27^$^	26.6^$^	48	49	30	28.2
ASA grade														
1 and 2	52.8	58.3	100	100			100	100	72.3	72.4	11	13	20	17.7
3 and above	47.2	41.7	0	0			0	0	27.7	27.6	89	87	80	83.3
Hypoxemia	0	19.4	2	21.6	21.2	33.1	0	8.4	9.4	33.5	39.3	45.2	7.7	9.1
Need for airway intervention	0	30.6	2	17.6			0.8	31.9	19.9	58.5	53.6	51.6	23.1	28.8

*ERCP* endoscopic retrograde cholangiopancreatography, *EGD* esophagogastroduodenoscopy, *BMI* body mass index, *ASA* American Society of Anesthesiologists, *HFNC* High-flow nasal cannula oxygen, *SNC* standard nasal cannula

*Unless otherwise indicated; ^#^gastroscopy and colonoscopy; ^$^median value

### Sources of heterogeneity

We identified potential sources of clinical heterogeneity. The population included in the studies and the definition of hypoxemia were both sources of clinical heterogeneity. The population included high risk of hypoxemia subjects and relatively low risk of hypoxemia subjects (The definition of risk of hypoxemia had been detailed in the “Study selection and data extraction” section). The definition of hypoxemia varied among studies. Most included hypoxemia diagnosed with SpO_2_ < 90% [21–24, 26], two studies included hypoxemia diagnosed with SpO_2_ < 92% [27, 28]. In our subgroup analysis, we included studies in which hypoxemia was defined as SpO_2_ < 90%.

### Quality assessment

**Fig.2:**
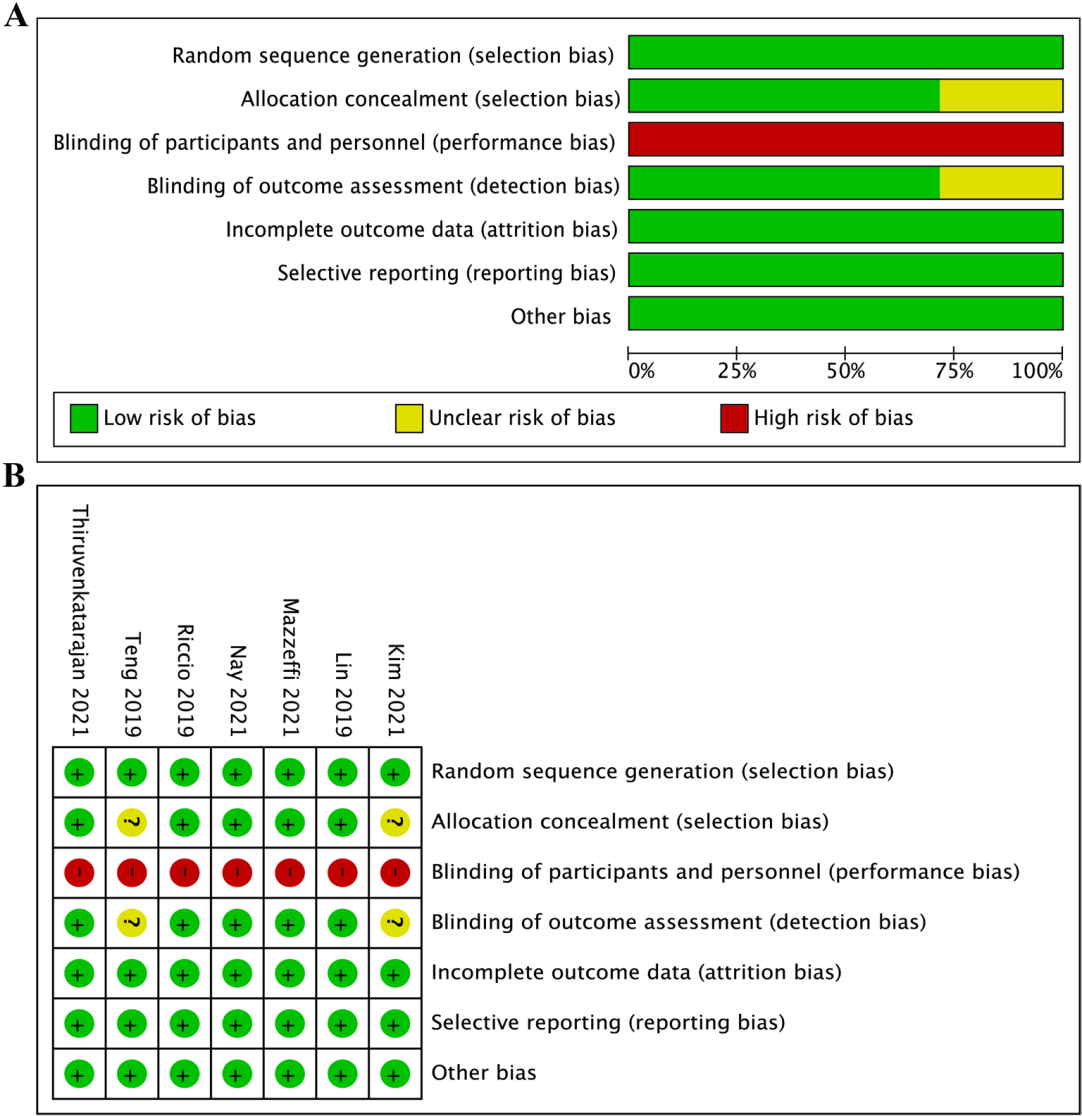
Risk of bias

The risk of bias of 7 included RCTs was evaluated based on the Cochrane Handbook. The risk of bias for each article was assessed, and the details of the results are presented in Fig. 2A, B. Because blinding of participants and personnel was impossible in these studies, it might lead to performance bias. Five studies reported appropriate allocation and concealment methods, but two studies did not describe specific strategies [22, 26]. Blinding of outcome assessment was unclear in two studies [22, 26], because they did not elaborate any methods to blind outcome assessors from group allocation. In terms of incomplete outcome data, 7 studies were at low risk of bias, for no data were missing. All seven studies had low risk in randomization of sequence generation and selective reporting. Other risk, including potential source of bias was not found in all included studies.

### Primary outcomes

**Fig.3:**
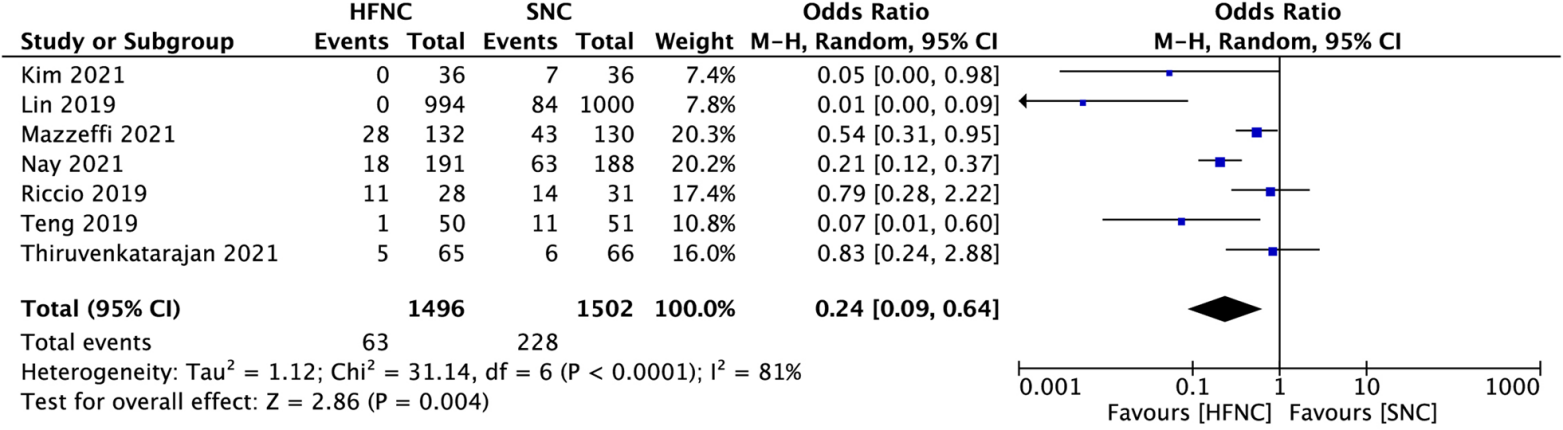
Comparison of hypoxemia incidence between high-flow nasal cannula oxygen (HFNC) and standard nasal cannula oxygen (SNC). CI confidence interval, M–H Mantel–Haenszel

**Fig.4:**
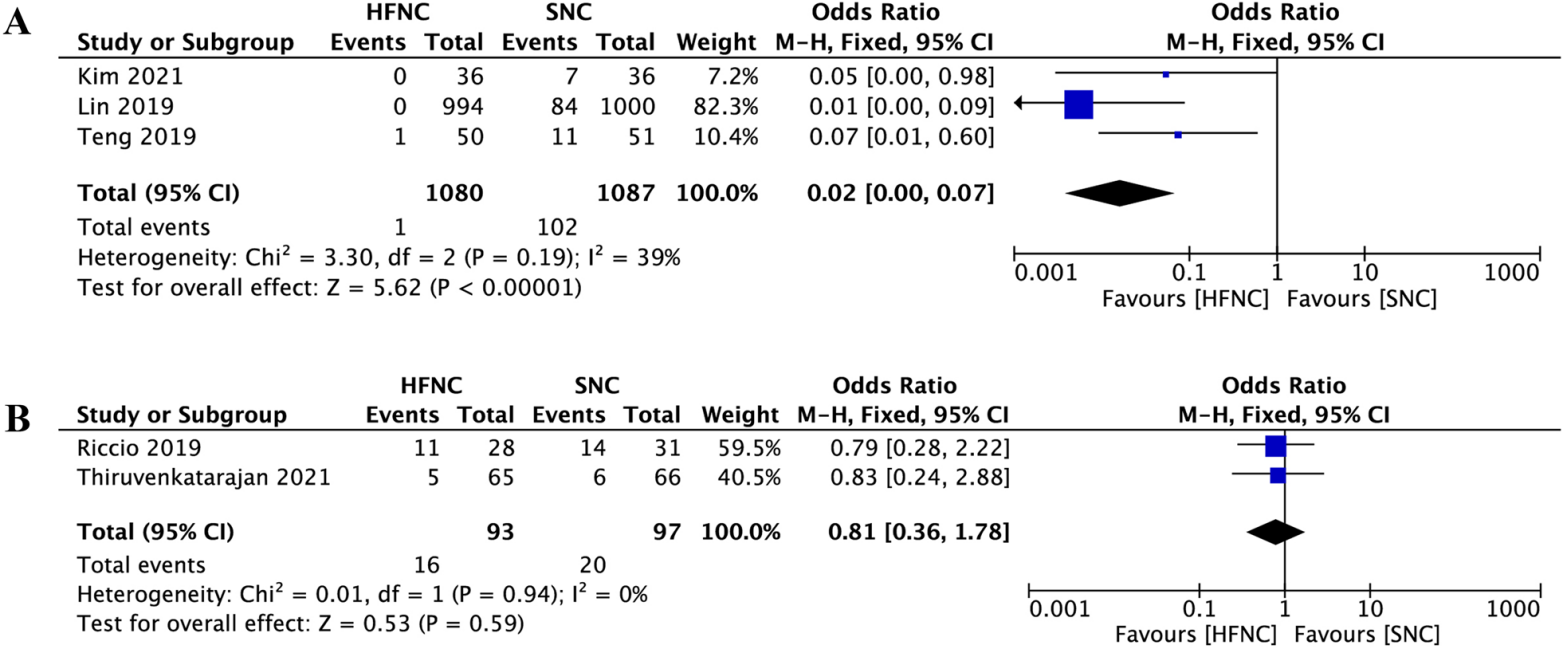
Subgroup analysis of hypoxemia incidence between high-flow nasal cannula oxygen (HFNC) and standard nasal cannula oxygen (SNC). hypoxemia was defined as SpO_2_ < 90%. **A** Low hypoxemia risk subjects; **B** High hypoxemia risk subjects. CI confidence interval, M–H Mantel–Haenszel

Compared to SNC, HFNC was associated with a significant reduction in hypoxemia incidence during sedated digestive endoscopy (OR 0.24, 95% CI 0.09 to 0.64, *P* = 0.004; M–H Random; *n* = 2998; heterogeneity I^2^ = 81%, *P* < 0.0001) (Fig. 3). In subgroup analysis, when hypoxemia was defined as SpO_2_ < 90%, low risk of hypoxemia subjects who received HFNC were associated with a significant reduction in hypoxemia incidence (OR 0.02, 95% CI 0.00 to 0.07, *P* < 0.00001; M–H Fixed; *n* = 2167; heterogeneity I^2^ = 39%, *P* = 0.19) (Fig. 4A). However, in the high risk of hypoxemia subjects, there was no difference between the two groups (OR 0.81, 95% CI 0.36 to 1.78, *P* = 0.59; M–H Fixed; *n* = 190; heterogeneity I^2^ = 0%, *P* = 0.94) (Fig. 4B).

### Secondary outcomes

**Fig.5:**
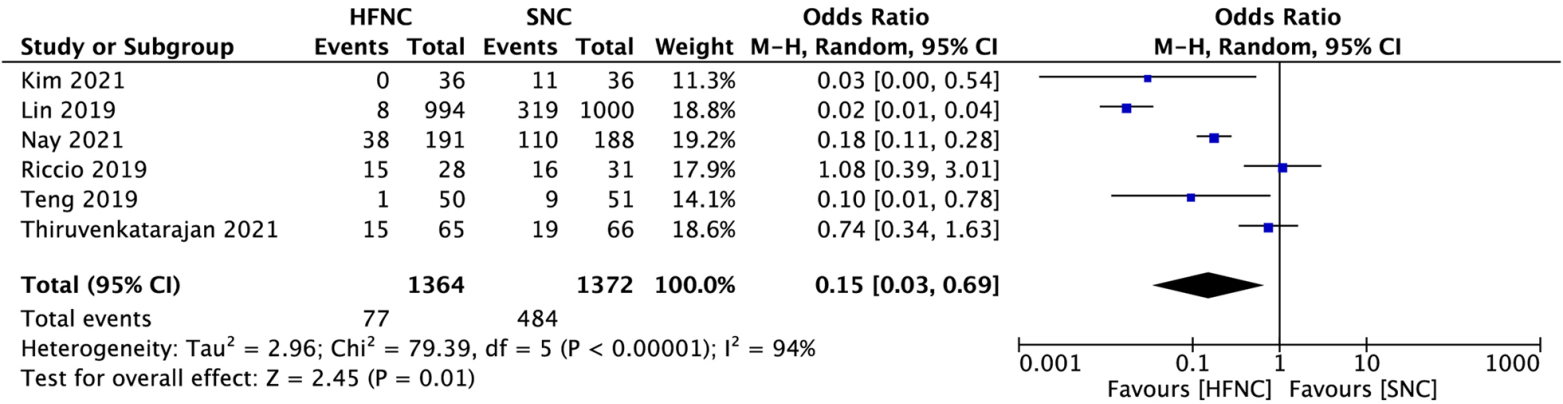
Comparison of airway intervention requirement between high-flow nasal cannula oxygen (HFNC) and standard nasal cannula oxygen (SNC). CI confidence interval, M–H Mantel–Haenszel

**Fig.6:**
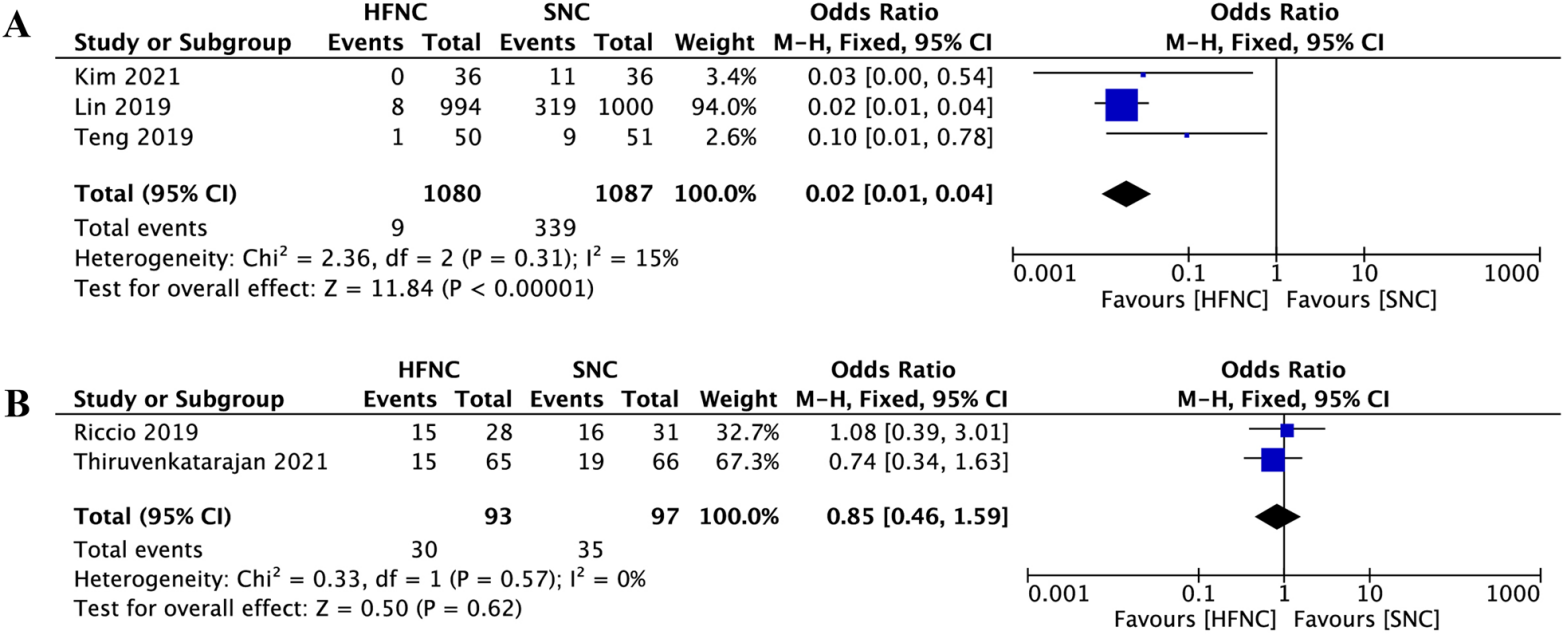
Subgroup analysis of airway intervention requirement between high-flow nasal cannula oxygen (HFNC) and standard nasal cannula oxygen (SNC). hypoxemia was defined as SpO2 < 90%. **A** Low hypoxemia risk subjects. **B** High hypoxemia risk subjects. CI confidence interval, M–H Mantel–Haenszel

When compared to SNC, pooled data suggested that the percentage of patients who need airway interventions during sedated digestive endoscopy was significantly decreased in the HFNC group (OR 0.15, 95% CI 0.03 to 0.69, *P* = 0.01; M–H Random; *n* = 2736; heterogeneity I^2^ = 94%, *P* < 0.00001) (Fig. 5). In subgroup analysis, when hypoxemia was defined as pulse oxygen saturation (SpO_2_) < 90%, low risk of hypoxemia subjects who received HFNC were associated with a significant reduction in airway intervention requirements (OR 0.02, 95% CI 0.01 to 0.04, *P* < 0.00001; M–H Fixed; *n* = 2167; heterogeneity I^2^ = 15%; *P* = 0.31) (Fig. 6A). However, in the high risk of hypoxemia subjects, there was no difference between the two groups (OR 0.85, 95% CI 0.46 to 1.59, *P* = 0.62; M–H Fixed; n = 190; heterogeneity I^2^ = 0%, *P* = 0.57) (Fig. 6B).

## Discussion

Hypoxemia is the most common negative effect of procedural sedation, and the rate of oxygen desaturation during sedated digestive endoscopy has been reported to be as high as 11–50% [29]. Although sedation-related mortality in digestive endoscopy is generally rare (about 0.08%), the harmful effect of hypoxemia is systemic, and even a short episode of hypoxemia to SpO_2_ lower than 90% is related to increased hospital length of stay, intensive care unit admissions and costs [30].

HFNC is a new oxygen administration technique for improving oxygenation and has been applied in many sedation procedures, such as sedated bronchoscopy and dental treatments [16, 20]. However, the use of HFNC in preventing hypoxemia during sedated digestive endoscopy has not been studied extensively. After our literature search, there are only 7 randomized controlled trials regarding the use of HFNC in digestive endoscopic sedation.

Consistent with previous studies in the sedated bronchoscopy and dental procedures [16, 20], we found that patients receiving HFNC were significantly less likely to desaturate than SNC in adult patients during sedated digestive endoscopy (Fig. 3). We also clarified the beneficial effect of HFNC therapy in reducing the requirement of airway maneuvers during sedation procedures (Fig. 5). We believe that the advantage of HFNC for preventing hypoxemia during sedated digestive endoscopy is mainly reflected in the following two aspects. First, by providing high and constant oxygen flow, HFNC overcomes the issue of room air entrainment [31]. FiO_2_ is more consistent at higher flows and can be maintained as high as 100% [32, 33]. A high fraction of inspired oxygen is undoubtedly helpful for preserving pulse oxygen saturation. However, maintaining a hundred percent fraction of inspired oxygen via a standard nasal cannula is impossible. Even with the highest oxygen flow rate, the maximal FiO_2_ cannot exceed eighty percent [34]. Second, depending on the high flow, HFNC can flush out the anatomical dead space and supply positive airway pressures without increasing the volume of gastric secretions [35]. These effects facilitate both oxygenation and carbon dioxide clearance during sedation [36], when inspiratory efforts may be somewhat suppressed.

Although the overall hypoxemia incidence and airway intervention requirements of the HFNC group were significantly lower than that of the SNC group, data of the included studies showed high heterogeneity (Primary outcome: I^2^ = 81%; Secondary outcome: I^2^ = 94%) (Figs. 3, 5). We tried to identify potential sources of clinical heterogeneity and made a subgroup analysis. We found that populations enrolled in these seven studies were different. In three of these articles [37–40], the subjects included were obese (BMI > 30), or had obstructive sleep apnoea syndrome, or were ASA physical status 3 or 4, which may all contribute to the development of hypoxemia during sedation for gastrointestinal endoscopy. Four other studies enrolled subjects were of relatively low risk for developing hypoxemia, such as with more people BMI < 30, or were ASA physical status 1 or 2. It seems acceptable to explain the heterogeneity from the perspective of hypoxemia risk during digestive endoscopic sedation, because populations with different hypoxemia risks may respond differently to HFNC [23, 41, 42]. Besides, we also note that hypoxemia in these studies was inconsistently defined. Most included hypoxemia defined as a SpO_2_ of < 90%, and two studies included hypoxemia defined as a SpO_2_ of < 92%. The SpO_2_ readings of 90% to 94% are often defined as arterial oxygen desaturation, while a threshold of SpO_2_ < 90% is used to define arterial hypoxemia. Different SpO_2_ thresholds may account for different study results [43, 44]. Therefore, in our further analysis, we defined hypoxemia as SpO_2_ lower than 90%, and with that definition, we made a subgroup analysis based on populations with different hypoxemia risks.

In subgroup analysis, compared to SNC, patients at low risk of hypoxemia who received HFNC were associated with a significant reduction in hypoxemia incidence and airway intervention requirements with both low heterogeneities (Figs. 4A and 6A). However, in the high risk of hypoxemia patients who underwent sedated digestive endoscopy, there was no difference between the two supportive oxygen therapy methods in both primary and secondary outcomes with both low heterogeneities (Figs. 4B and 6B). In the high risk of hypoxemia patients, the effectiveness of HFNC during sedated digestive endoscopy was not superior to the SNC, and there are two possible reasons. First, subjects enrolled in this group were either morbidly obese or with high ASA physical status, and they were more likely to face varying degrees of hypoventilation under sedation [45]. Although HFNC can provide higher FiO_2_ than SNC, it is unlikely to ameliorate hypoxaemia developing from sedation induced hypoventilation or shunt [16, 46, 47]. Second, to overcome hypoventilation, patients at high risk of hypoxemia often need more positive airway pressures during sedation. With mouth closed and a maximum flow of 60 L/min, positive airway pressure generated by HFNC therapy may reach up to 5.6 cmH_2_O [48]. However, a previous study showed that the conscious patient breathes mostly nasally while the sedated patient breathes mostly orally during esophagogastroduodenoscopy [49]. The opening of the mouth during digestive endoscopic sedation causes the escape of HFNC gas, which reduces the positive airway pressure to 1.7 cmH_2_O [48]. Such a small airway pressure has little effect on preventing hypoxemia in these patients. Therefore, the greatest challenge of providing adequate oxygenation in high-risk patients undergoing sedation is the inability to provide appropriate positive airway pressures. In recent years, by creating new types of masks or ventilation systems, device manufacturers have stepped in to fill this void, and some masks such as endoscopic nasal mask [50] and SuperNO2VA™ nasal PAP ventilation system [51] had been proven useful in these high-risk patients during sedated digestive endoscopy.

## Limitations

Although this is the first systematic review and meta-analysis evaluating the effects of HFNC in digestive endoscopic procedures under sedation, some limitations of our study need to be noted. First, the flow rate of HFNC, the fraction of inspired oxygen, patient's positioning, sedation agents, the depth of sedation, and duration of endoscopy ranged widely in our enrolled studies, which may further impede the clinical practice. Second, although in the subgroup analysis of high risk of hypoxemia patients, we found HFNC showed no better outcomes, the sample size of included studies was small. More RCTs with large sample size were needed to demonstrate convincing results. Furthermore, in the subgroup analysis of low risk of hypoxemia patients; because of the relatively large sample size, Lin's [21] study may be overpowering the other studies. However, even if it overpowers or is heavily weighted, our meta-analysis or that single study itself still suggest the potential benefit of using HFNC in low-risk patients which decreases the risk of hypoxemia or need for interventions. Third, bias cannot be completely ruled out, because blinding of participants and personnel was impossible.

## Conclusions

This systematic review and meta-analysis suggest that HFNC is more effective than SNC in preventing hypoxemia and avoiding airway interventions during sedated digestive endoscopy, but the subgroup analysis shows that HFNC may be more suitable for patients at low risk of hypoxemia.

## Supplementary Information

**Additional file 1:** Search strategy.

## Data Availability

All data generated or analyzed during this study are included in this published article.

## Abbreviations

ERCP: Endoscopic retrograde cholangiopancreatography
EGD: Esophagogastroduodenoscopy
BMI: Body mass index
ASA: American Society of Anesthesiologists
HFNC: High-flow nasal cannula oxygen
SNC: Standard nasal cannula
OR: Odds ratio
CI: Confidence interval
M–H: Mantel–Haenszel
