# Supplementary material for: The effectiveness of high-flow nasal cannula during sedated digestive endoscopy: a systematic review and meta-analysis
Source: Eur J Med Res. 2022 Feb 24;27:30. doi: 10.1186/s40001-022-00661-8 (PMC8876126; doi:10.1186/s40001-022-00661-8)
Supplement: Supplementary file 1 — Additional file 1: Search strategy. [file 40001_2022_661_MOESM1_ESM.docx]

Supplementary Material

**Search strategy**

| Number | Searched for |
| --- | --- |
| #1 | “digestive endoscopy” OR “endoscopic retrograde cholangiopancreatography” OR “ERCP” OR “gastrointestinal endoscopy” OR “esophagogastroduodenoscopy” OR “EGD” OR “gastroscopy” OR “colonoscopy” OR “endoscopy” OR “endoscopic procedure” |
| #2 | (“High Flow” AND Nasal) OR (“High Flow” AND Therapy) OR (“High Flow” AND "Oxygen") OR “HFNC” OR “HHFNC” OR “HHHFNC” OR “NHF” OR “humidified high-flow nasal cannula” OR “heated humidified high-flow nasal cannula” OR “HHHFNC” OR “HFNO” OR “NHF” OR “high flow nasal oxygen” OR “high flow nasal cannula” |
| #3 | “sedation” OR“midazolam” OR “benzodiazepines” OR “propofol” OR “anesthesia”  OR “fentanyl” OR “lidocaine” OR “opioids” OR “alfentanil” |
| #4 | “Randomized Controlled Trial” OR “Controlled Clinical Trial” OR “Randomized” OR “Randomly” OR “RCT” |
| #5 | #1 and #2 and #3 and #4 |
